# P-821. Predictive Value of MRSA Nares PCR in Patients Hospitalized for More than Seven Days

**DOI:** 10.1093/ofid/ofaf695.1029

**Published:** 2026-01-11

**Authors:** Rebecca O’Toole, Karrine Brade, Tyree H Kiser

**Affiliations:** UCHealth- University of Colorado Hospital, Denver, CO; University of Colorado Hospital, Aurora, Colorado; University of Colorado Skaggs School of Pharmacy and Pharmaceutical Sciences, Aurora, Colorado

## Abstract

**Background:**

Methicillin-resistant *Staphylococcus aureus* (MRSA) nares polymerase chain reaction (PCR) screening tests have a high negative predictive value (NPV) for MRSA pneumonia. They are an effective antimicrobial stewardship tool often used to guide the use of anti-MRSA agents for the treatment of pneumonia. Previous studies evaluating the predictive values of the MRSA nares PCR focus on testing within 48-72 hours of hospital or intensive care unit admission, but a paucity of literature exists regarding the predictive values throughout admission.

**Methods:**

This retrospective single cohort study evaluated the predictive value of MRSA nares PCR screening completed after seven days into hospital admission. All patients admitted between January 2023 and December 2023 with pneumonia, an MRSA nares PCR test done at least seven days after admission, and respiratory cultures done within 72hours after the MRSA nares PCR were included. Results of the MRSA nares PCR and respiratory cultures were used to calculate the sensitivity, specificity, positive predictive value (PPV) and NPV for MRSA pneumonia.

**Results:**

Ninety-seven unique encounters were included, representing ninety-six patients total. The MRSA nares PCR was drawn a mean of 17.2 days into admission and positive in 7.22% (7/97) of cases. Respiratory cultures confirmed MRSA pneumonia in 4.12% (4/97) of cases. The MRSA nares PCR demonstrated a sensitivity of 100%, a specificity of 95.7%, a PPV of 33.3% and a NPV of 100%. Only 1 patient had MRSA PCR conversion from negative to positive in the cohort. Furthermore, the results were similar for patients who had received at least seventy-two hours of anti-MRSA therapy with vancomycin, linezolid, daptomycin, clindamycin or ceftaroline prior to their MRSA PCR test, with a NPV of 100%.

**Conclusion:**

This data suggests that the predictive values of MRSA nares PCR are not significantly impacted by length of stay or exposure to anti-MRSA therapy. Additionally, the negligible MRSA nares PCR conversion rate supports the use of these tests completed upon admission to guide therapy throughout the encounter and that repeated screenings during prolonged hospital admissions may be unnecessary.

**Disclosures:**

All Authors: No reported disclosures

